# Isolation of Human Photoreceptor Precursors via a Cell Surface Marker Panel from Stem Cell‐Derived Retinal Organoids and Fetal Retinae

**DOI:** 10.1002/stem.2775

**Published:** 2018-02-01

**Authors:** Jörn Lakowski, Emily Welby, Dimitri Budinger, Fabiana Di Marco, Valentina Di Foggia, James W.B. Bainbridge, Kyle Wallace, David M. Gamm, Robin R. Ali, Jane C. Sowden

**Affiliations:** ^1^ Stem Cells and Regenerative Medicine Section, University College London London United Kingdom; ^2^ NIHR Great Ormond Street Hospital Biomedical Research Centre, UCL Great Ormond Street Institute of Child Health, University College London London United Kingdom; ^3^ Department of Genetics UCL Institute of Ophthalmology London United Kingdom; ^4^ Waisman Center, University of Wisconsin‐Madison School of Medicine and Public Health, Waisman Center Rm T609 Madison Wisconsin USA; ^5^ Department of Ophthalmology and Visual Sciences and McPherson Eye Research Institute University of Wisconsin‐Madison School of Medicine and Public Health, Waisman Center Rm T609 Madison Wisconsin USA

**Keywords:** Retina, Retinal photoreceptors, Cell surface markers, Stem cell culture, Embryonic stem cells, Induced pluripotent stem cells

## Abstract

Loss of photoreceptor cells due to retinal degeneration is one of the main causes of blindness in the developed world. Although there is currently no effective treatment, cell replacement therapy using stem‐cell‐derived photoreceptor cells may be a feasible future treatment option. In order to ensure safety and efficacy of this approach, robust cell isolation and purification protocols must be developed. To this end, we previously developed a biomarker panel for the isolation of mouse photoreceptor precursors from the developing mouse retina and mouse embryonic stem cell cultures. In the current study we applied this approach to the human pluripotent stem cell (hPSC) system, and identified novel biomarker combinations that can be leveraged for the isolation of human photoreceptors. Human retinal samples and hPSC‐derived retinal organoid cultures were screened against 242 human monoclonal antibodies using a high through‐put flow cytometry approach. We identified 46 biomarkers with significant expression levels in the human retina and hPSC differentiation cultures. Human retinal cell samples, either from fetal tissue or derived from embryonic and induced pluripotent stem cell cultures, were fluorescence‐activated cell sorted (FACS) using selected candidate biomarkers that showed expression in discrete cell populations. Enrichment for photoreceptors and exclusion of mitotically active cells was demonstrated by immunocytochemical analysis with photoreceptor‐specific antibodies and Ki‐67. We established a biomarker combination, which enables the robust purification of viable human photoreceptors from both human retinae and hPSC‐derived organoid cultures. Stem Cells *2018;36:709–722*

**Significance Statement**

Retinal differentiation of human pluripotent stem cells provides a renewable source of photoreceptor cells for use in a cell replacement therapy for retinal degeneration and for the study of retinal development and disease. However, a robust method of purifying photoreceptors remains to be determined. This study defines a combination of cell surface marker monoclonal antibodies that can be used in a fluorescence‐activated cell sorting approach to significantly enrich for human photoreceptor cells. This strategy isolates photoreceptors without the need for genetic modification or the addition of reporter genes, which is a critical requirement for developing clinical therapies

## Introduction

Rod and cone photoreceptors, which are located in the outer nuclear layer within the retina, are the primary sensory cells of the mammalian visual system. Stimulation by light results in the initiation of the visual transduction cascade, whose information is relayed to bipolar cells, processed by horizontal and amacrine interneurons, and eventually sent by retinal ganglion cells to the visual processing centers in the brain 1. As the mammalian nervous system has limited regenerative capacity, any disease or injury to retinal photoreceptors leads inevitably to visual impairment or even blindness. In industrialized countries, degenerative conditions affecting photoreceptors, such as retinitis pigmentosa (RP) 2, age‐related macular degeneration 3, or Leber's congenital amaurosis 4, are main causes of blindness and their incidences are expected to rise over the next decades due to an increase in life expectancy. There is currently a paucity of effective treatments for retinal degeneration 5, 6 and cell replacement therapy may offer a therapeutic approach for patients with advanced disease where there has been extensive photoreceptor loss 7, 8, 9. Importantly, while rod and cone photoreceptor death proceeds, the internal retinal architecture remains unaltered for some time, providing a window of opportunity to re‐introduce light‐sensing cells via cell transplantation 7. Cell replacement has been the subject of intense investigation, and studies from several research groups suggest that some visual function can be re‐established in blind mice after subretinal transplantation of post‐mitotic photoreceptor precursor cells 10, 11, 12. While other cell types have been tested, including neural and retinal progenitors 13, as well as fully mature photoreceptors 14, the most feasible donor cell type thus far has been the immature, but no longer dividing rod or cone precursor cells 15, 16, 17, 18, possibly owing to their ability to survive purification procedures and differentiate in the host retina 10.

One of the main hurdles for the development of a retinal cell replacement therapy is the need for a sustainable and consistent donor cell source. Currently, the most promising cell sources for photoreceptor replacement therapy are embryonic or induced pluripotent stem cells, that have the ability to not only self‐renew but also to generate, most, if not all cell types of the human body, including photoreceptors 19, 20, 21, 22, 23. In particular, the development of three‐dimensional retinal organoid culture systems, which recapitulate retinal histogenesis in a time frame consistent with normal development, has been a major advance toward the production of appropriate donor cell populations for human retinal cell therapy 20, 21, 22, 23. However, while significant progress has been made in the refinement of retinal culture methods, all protocols thus far result in the production of a range of different cell types, retinal and otherwise, at different stages of development. It is therefore critical to develop methods to extract and purify potential donor cells from these heterogeneous mixtures of cells in order to maximize safety and efficacy in the clinical setting. One particular concern is the removal of mitotically active cells from donor cell preparations, which may otherwise lead to tumor formation post‐transplantation 24. We previously developed a cell isolation method based on the use of CD (cluster of differentiation) antibodies effective for the isolation of transplantation‐competent photoreceptor precursors from mouse embryonic stem cell‐derived organoid cultures 16, 17. In this study, we developed a cell surface biomarker panel that can be leveraged for this isolation of human photoreceptor cells from hPSC differentiation cultures.

## Materials and Methods

### Animals

Experimental mice were kept in University College London animal facilities and all experiments were conducted in agreement with the Animals (Scientific Procedures) Act 1986 and the Association for Research in Vision and Ophthalmology Statement for the Use of Animals in Ophthalmic and Vision Research. *C57Bl/6J,* and *C3H/HeNCrl* (RD1; *Pde6b* ^*rd1/rd1*^) recipient mice at 3 weeks of age at the time of transplantation were obtained from Charles Rivers laboratory.

### Human Tissue

The human embryonic and fetal material was provided by the Joint Medical Research Council U.K. (Grant G0700089)/Wellcome Trust (Grant GR082557) Human Developmental Biology Resource (http://hdbr.org). All work was carried out with ethics committee approval and in line with UCL and Human Tissue Authority codes of practice under HDBR license.

### Human Pluripotent Stem Cell Culture

All pluripotent stem cells were maintained as previously described for hESC cultures. Briefly human embryonic stem cells (WiCell H9), and induced pluripotent cells (NCUS:7 (WTN7), NCUS:24) were either cultured under feeder‐free conditions using mTesR1 (Matrigel; BD Biosciences), or on a feeder layer of irradiated mouse embryonic fibroblasts (MEFs; GlobalStem) in embryonic stem cell medium (DMEM/F12 (1:1), 20% knockout serum replacement, 0.1 mM mercaptoethanol, 1 mM l‐glutamine, MEM nonessential amino acids, and 4 ng/ml FGF2), respectively. For feeder‐dependent culture, hiPSCs were passaged every 5–6 days using Dispase and Collagenase, and morphologically identifiable differentiated cells were mechanically removed at each passage. hESCs cultured in mTeSR1; Stemcell Technologies on matrigel substrate were passaged with RelesR; Stemcell Technologies reagent as recommended by the manufacturer. human induced pluripotent stem cell (hiPSC) and hESC lines used for this study showed comparable overall generation (17.1% ± 13.7% vs. 16.1% ± 11%, respectively), and enrichment (61.7% ± 17% vs. 60.8% ± 14%) of CRX+/RECOVERIN+ photoreceptors (Supporting Information Fig. S1).

### Retinal Differentiation of hESC and hiPSC Cultures

The method for differentiating hPSC cells toward a retinal fate was carried out according to a previously described protocol 21 but with minor modifications. The differentiation time line and output were approximately the same between different cell lines and independent of maintenance conditions (Supporting Information Fig S1). Briefly, hPSC cultures were lifted enzymatically using dispase (1 mg/ml) and grown as aggregates/embryoid bodies in EB media for 4 days without FGF2. Embryoid bodies were then transferred to a defined neural induction medium, which consisted of DMEM/F12, 1% N2 supplement, 1% MEM nonessential amino acids, 1% Glutamax, and 2 µg/ml heparin. At day 6 of differentiation, EBs were allowed to settle in laminin (LN2020) coated tissue culture plates (6 well) and remained in this configuration for the remainder of the experiment. After 10 days post lifting, neuroepithelial structures were visible within the cultures. On day 16, cultures where switched to chemically‐defined retinal differentiation media consisting of DMEM, F12 (3:1) supplemented with 2% B27. In contrast to the originally described protocol, optic vesicle‐like structures were not lifted by cutting, but remained in the same culture vessel.

### Human Retinal Cultures

Human fetal retinal tissue was microdissected and dissociated using a papain solution according to manufacturer's recommendation (Worthington Biochemical Corporation, Lorne Laboratories, U.K.). Cells were seeded on poly‐l‐lysine (Sigma‐Aldrich) and laminin (Sigma‐Aldrich, 1 mg/ml) coated glass coverslips and cultured in retinal differentiation media containing DMEM‐F12 Glutamax (Invitrogen), 1 x N2 and 1 x B27 neural supplements (Invitrogen), and 10% fetal bovine serum (FBS) (Invitrogen) as well as penicillin/streptomycin (Invitrogen). Cell culture media was changed every 2–3 days.

### Histology and Immunohistochemistry

Retinal organoid specimens or human fetal retinae were fixed in 4% (wt/vol) phosphate‐buffered formaldehyde solution at 4°C for 15–30 minutes, washed three times with phosphate‐buffered saline (PBS) and equilibrated in 30% (wt/vol) sucrose solution at room temperature for 1 hour. Specimens were then transferred into an optimal cutting temperature (OCT)‐compound (RA Lamb) and frozen in a dry ice‐methylbutane slurry. A Leica CM1900UV cryostat was used to produce 18 μm thick sections, which were collected onto Superfrost plus glass slides (VWR). For immunochemical analysis OCT compound was removed by a 15‐minutes incubation in 37°C PBS and cryosections were blocked with 10% (vol/vol) FBS, 1% (wt/vol) bovine serum albumin (BSA) in PBS containing 0.1% (vol/vol ) Triton X‐100 for one hour at room temperature. The following primary antibodies were used in the same blocking solution for 1 hour at room temperature of 4°C overnight; Recoverin, Millipore, 1:1,000; Ki67, Abcam, 1:300; CRX (clone 4G11), Sigma, 1:1,000; Pax6, Covance, 1:1,000; RAX, Abcam, 1:1,000; OTX2, Abcam, 1:200; BRN3b, Millipore/Upstate; 1:300; AP2a, Hybridoma Bank, 1:100.

The primary antibody was omitted for negative controls. Primary antibody staining was followed by three washes with 1x PBS. Subsequently, cryosections were incubated for 1 hour at room temperature with the appropriate secondary antibody diluted in blocking solution (Goat anti‐rabbit Alexa Fluor 594, Invitrogen, A‐11037, Goat anti‐mouse Alexa Fluor 594, Invitrogen; all 1:800). Hoechst 33342 (1:3,000, Sigma‐Aldrich) was applied for 10 minutes at room temperature to counterstain nuclei, followed by three washes with PBS prior to cover‐slipping with the Citifluor AF‐1 (Electron Microscopy Science) mounting medium.

### Dissociation of Human Retinae/hPSC Organoid Cultures and Flow Cytometry

Human retinae or hPSC‐derived retinal organoids were isolated via microdissection and dissociated into a homogenous single cell suspension using a papain based, enzymatic method according to manufacturer's instructions (Worthington Biochemical, Lorne Laboratories, U.K.). Fetal human eyes ranged in age from 10 to 22 post conception weeks, whereas hPSC differentiation cultures were harvested at either day 100 or day 200. Dissociated cells were resuspended in flow‐cytometry blocking buffer (1%BSA, PBS) and kept on ice for 30 minutes. Subsequently, conjugated, monoclonal flow antibodies were added to the samples (1 × 10^6^ cells in 100 μl) and incubated for 1 hour, protected from light. The following monoclonal antibodies were used for FACS‐analysis (LSRII) and FAC‐sorting (BD FACS AriaIII) as recommended by supplier: CD73‐PECy7, BD; CD133‐PE, Miltenyi; CD29‐BV510, BD; SSEA‐1APC, BD; CD90‐APC, BD; CD9‐APC, BD; CD200‐V450; CD49f‐BV650; EGFR1‐PE, BD; GD2‐PE, BD; CD184‐PE, BD; SSEA‐4‐PerCP‐Cy5.5. After staining the cells were centrifuged at 300*g* for 5–10 minutes at 4°C and resuspended in FACS blocking buffer and kept on ice until use. FACS gates were defined according to isotype controls where available and more than 10,000 cells analyzed. Compensations were applied using BD FACSDiva software using singly stained control samples. Data presented is from at least 3 independent replicates.

### Immunocytochemistry on Dissociated and FAC‐Sorted hESC‐Derived and Fetal Retinal Cells

hPSC‐derived retinal organoid cultures or fetal human retinae (10–22 pcw) were dissociated and sorted via the biomarker panel as described above. Post sort cells were spun down at 300*g* for 15 minutes at 4°C and plated on poly‐lysine/laminin coated chamber slides (Labtec) and allowed to adhere for 30 minutes at 37°C. Chambers were then washed once with PBS and adherent cells fixed with 4% PFA/PBS for no more than 10 minutes at room temperature. Following three times washing with PBS, samples were blocked in 10% FBS, 1% BSA, 0.1% (vol/vol) Triton X‐100 in PBS for 1 hour at room temperature. The blocking solution was replaced with staining solution containing primary antibody in in 10% FBS, 1% BSA, 0.1% (vol/vol) Triton X‐100 in PBS. The primary antibody was omitted for negative controls. Finally chambers with adherent cells were incubated for 1 hour at room temperature with the secondary antibody diluted in blocking solution (Invitrogen, Goat anti‐rabbit Alexa Fluor 594; Goat anti‐mouse 488) and counter stained for 5 minutes with DAPI (Sigma‐Aldrich). The percentage of positive cells in the experimental groups was established by cell counter function, using confocal tile scans; > 100 cells were counted from three biological replicates for each condition. Individual differentiation experiments for each hPSC cell line were analyzed as separate data sets. As the mean values, as well as standard deviation, for the cell lines were similar (Supporting Information Fig. S1), outcomes from the photoreceptor enrichment assays were aggregated to obtain the mean enrichment across different cell lines. Similarly, output from enrichment experiments using fetal material was combined except where indicated. All enrichment values are given as mean ± standard variation. ANOVA was used for statistical analysis.

### BD Lyoplate Antibody Screen

Human fetal, post‐mortem adult and day 90 hPSC‐derived retinal organoids (hiPSC line NCUS:7) were harvested and dissociated to single cell suspensions as described above. For BD lyoplate screens we followed the manufacturer's recommendations. All centrifugation steps were carried out at 300*g* for 5 minutes at 4°C. After dissociation, retinal cells were resuspended in BD FACS staining buffer and adjusted to a cell concentration of 10 million cells per 1 ml followed by transfer of the cells into round bottom 96‐well plates (BD Falcon, Cat. No. 351177). Twenty microliters of reconstituted primary antibody solution was then added to the cells, mixed and incubated on ice for 30 minutes. This was followed by several washing steps with FACS staining buffer (BD Pharmingen) after which the cells were incubated for 30 minutes with the appropriate biotinylated secondary antibody. Following several washes, 100 μl of Alexa Fluor 647 Streptavidin (1:4,000, 0.5 μg/ml) was added to each well containing cells stained with the biotinylated secondary antibodies and incubated on ice in the dark for 30 minutes. Stained cells were then washed three times and analyzed on a BD FACSCalibur. At least 30,000 events were collected for the analysis using FACSDiva software and FlowJo.

### Retinal Cell Transplantations

For transplantation, cells were incubated in blocking solution (1% BSA/PBS) for 1 hour and subsequently stained with specific monoclonal antibodies directed toward CD29 and SSEA‐1, or respective isotype controls, according to manufacturer's recommendations. Photoreceptor precursors were enriched by FAC‐sorting (BD FACS AriaIII) with gating determined using single stained controls and combined isotype controls. Cells in experimental group “unsorted” were processed identically to labeled cells except they were ungated. Post sort cell viability was > 85% based on DAPI staining, and the sorted cells were resuspended at 100,000 live cells per μl in injection buffer (EBSS, DNaseI) after centrifugation at 300*g* for 15 minutes using a Heraeus Labfuge 400R (Thermos, U.K.).

Recipient mice (4 weeks, C57Bl/6J or *C3H/HeNCrl*, RD1) were anaesthetized with an intraperitoneal injection of 0.2 ml of a mixture of Domitor (1 mg/ml (medetomidine hydrochloride, Pfizer Pharmaceuticals, Kent U.K.), ketamine (100 mg/ml, Fort Dodge Animal Health, Southampton, U.K.), and sterile water (ratio 5:3:42). One percent tropicamide solution was applied to dilate pupils of animals and injections were performed using a Zeiss operating microscope. Fundi were visualized using a contact lens system consisting of a coverslip and a drop of coupling medium liquid (Viscotears, Novartis Pharmaceuticals, Frimley, U.K.). One microliter of cell suspension (containing 100,000 live FAC‐sorted cells) was injected via a 34G needle under direct visualization through the superior equatorial sclera and guided into the subretinal space and toward the posterior pole, creating a self‐sealing sclerotomy. Following injection, anesthesia was reversed by intraperitoneal administration of 0.2 ml of Antisedan (atipamezole hydrochloride 0.10 mg/ml, Pfizer, Kent U.K.). The retinas of recipient mice were harvested from three transplantations and processed for analysis.

### Microscopy, Image Acquisition, and Processing

A Zeiss LSM710 (Zen2009, Zeiss) was used for acquisition of confocal images. Images were processed in Zen2009 (Zeiss), Photoshop CS4 (Adobe), Illustrator CS4 (Adobe), and FIJI. Double‐labeling analysis was carried out in Adobe Photoshop CS4.

### Transcript Analysis Reverse Transcription Polymerase Chain Reaction

Total RNA was extracted from unsorted or FAC‐sorted human cell populations using the RNeasy Mini Kit (Qiagen, U.K.). An on‐column DNA digest was carried out to eliminate all trace amounts of genomic DNA from the samples. Following quantification of total RNA using a NanoDrop ND‐1000 spectrophotometer, cDNA was generated by means of M‐MLV‐reverse transcriptase (Promega). The following primers were used for reverse transcription polymerase chain reaction (RT‐PCR) using Bioline Taq polymerase: SIX6, For AGCTGCCCATCTTGAATTTCAG, Rev TCAGATGTCGCACTCGCTG; RX, For TCAGAGGAGGAACAGCCCAAGAAA, Rev TCATGGAGGACACTTCCAGCTTCT; VSX2, For ATTCAACGAAGCCCACTACCCAGA, Rev ATCCTTGGCTGACTGAGG‐ATGGA; PAX6, For CCAGGCAGAGCCAGCATGCAGAACA, Rev GGTTGGTAGA‐CACTGGTGCTGAAACT; RECOVERIN, For CTGCCCACTCTTCCTCACTC, Rev CAAACTGGCATCAGTCGC‐AGA; CRX, For CTATTCTGTCAACGCCTTGGC, Rev CTATTCTGTCAACGCCTTGGC; MITF, TTCACGAGCGTCCTGTATGCAGAT, Rev TTGCAAAGCAGGATCCACAAGCC; OTX2, For CAGTCAATGGGCTGAGTCTG‐AC, Rev GACAGTGGGGAGATGGAAGC; GAPDH, For CCTTCATTGACCTCAACT‐ACA, Rev CTAAGCAGTTGGTGGTGCAGG.

## Results

### Identification of Cell Surface Biomarkers Expressed in the Developing and Mature Human Retina and hPSC‐Derived Organoid Cultures

**Figure 1:**
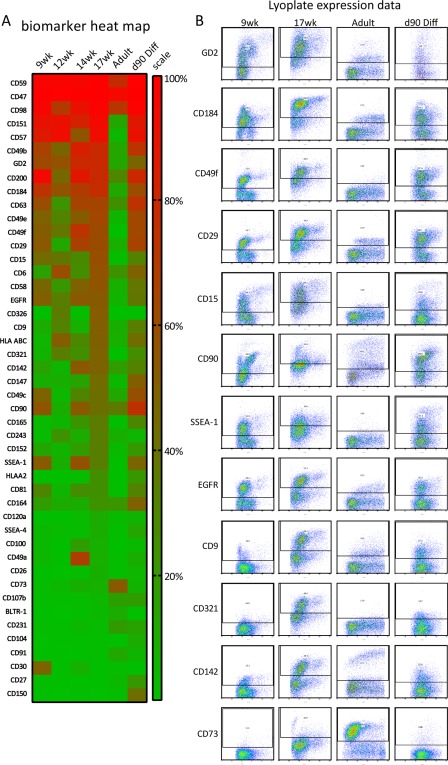
Lyoplate antibody screen using human retinal tissue. **(A)**: Heat map expression summary of top 46 cluster of differentiation (CD) biomarkers (*Y*‐axis) identified in human retinal tissue and human pluripotent stem cell‐derived retinal organoid cultures day 90 of differentiation (d90 Diff; human induced pluripotent stem cell (hiPSC) line NCUS:7). (*X*‐axis). The scale indicates the percentage of cells labeling with each marker (red = high; green = low). **(B)**: Flow cytometry traces of CD biomarkers (*Y*‐axis) labeling distinct retinal cell populations during development of the human retina. *X*‐axis represents forward scatter.

We sought to identify a human CD (cluster of differentiation) biomarker set useful for the isolation of human photoreceptors taking advantage of fetal (9, 12, 14, and 17 post conception weeks; PCW) and post‐mortem adult retinae. To this end, we screened dissociated whole retinal tissue against 242 well characterized human CD antibodies using the BD lyoplate system as a flow‐cytometry high‐throughput screening platform. We found 46 biomarkers that were expressed in human retinae at the various stages screened displaying substantial and robust labeling including many that delineated particular cell populations suggesting that they were either cell type or stage specific (Fig. 1). Some of these markers may be useful for the isolation of nonphotoreceptor cells, however, in this study, we focused our efforts on identifying either single or combinations of biomarkers useful for the enrichment of photoreceptors.

### Generation of Retinal Organoids Using hPSC Cultures

*Bone fide* human fetal and adult retinae are the gold standard tissue source for studying the development of the visual system in the human context. As the physiological quality of these samples can be variably problematic due to the nature of the tissue collection procedure (termination or post‐mortem), human pluripotent stem cell‐derived retinal organoid cultures represent a useful model system to generate human retinal cell types in a time frame consistent with normal retinal development, and provide a cell source applicable for cell replacement therapy.

For this study, we produced retinal organoids from human pluripotent stem cells utilizing a previously described differentiation protocol with some modification 8. Specifically, retinal organoids were kept adherent after neural induction, which not only reduced necessary handling, but could also allow facile scale up of the method toward cell production for cell replacement therapy as mechanical isolation of organoids by microdissection is not necessary. Using this approach photoreceptor precursors could be generated from both feeder‐dependent as well as feeder‐free culture systems with comparable efficiency (Supporting Information Fig. S1) (see Material and Methods for details).

**Figure 2:**
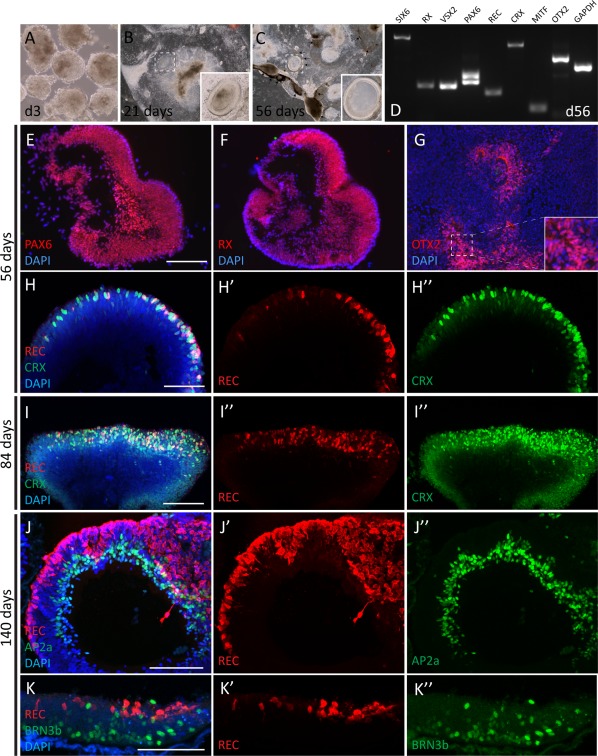
Stem cell‐derived retinal organoids. **(A–K)**: Generation of retinal tissue using hPSC cultures (iPSC). **(A‐C)**. Optic vesicle‐like structures are present from 21 days (insets); arrowheads indicate pigmentation. Retinal organoids containing the major retinal cell types are generated within a time frame consistent with human development. (D): Reverse transcription polymerase chain reaction analysis showing expression of retinal markers at day 56. Eye field markers such as OTX2, RX, and PAX6 are present at day 56 (E–G). RECOVERIN/CRX double positive photoreceptors (PRs) are observed at day 56 onward (H, I, J, K). Ganglion and amacrine cells are juxtaposed to PRs similar to the human retina (J, K). Scale bar 100 μm.

After 3 weeks of differentiation, optic vesicle‐like (OV) structures became visible (Fig. 2A–2C) within the cultures and pigmentation started appearing in the surrounding cells grown attached to the culture vessel. At 8 weeks in culture, OVs displayed signs of internal lamination and strong pigmentation was widespread across the culture dish (Fig. 2C). Transcript and protein analysis revealed that many of the key players of retinal commitment such as PAX6, VSX2, MITF, and SIX6 were strongly expressed in the differentiation cultures (Fig. 2D–2F). In addition, markers of photoreceptor differentiation, for example, CRX, RECOVERIN, and OTX2 were also detected at both transcript and protein levels (Fig. 2G, 2H–2H’’). In later stages of culture, CRX/RECOVERIN expressing photoreceptor precursors populated the outer aspects of retinal organoids in this culture system (Fig. 2H–2H’’, 2I‐2I’’) whereas AP2a expressing amacrine cells and BRN3b positive ganglion cells were restricted to the internal surface of the structures (Fig. 2J–2J’’, 2K‐2K’’ and Supporting Information Fig. S2). While retinal cell types were often organized in laminated organoids, substantial proportions of photoreceptors and other retinal neurons were located in patches throughout the culture dish, often surrounded by retinal pigmented epithelial cells (data not shown).

### Enrichment of Photoreceptors Cells from hPSC‐Derived Organoid Cultures Using Cell Surface Biomarkers

In order to identify human biomarkers useful for the enrichment of photoreceptors from human stem cell sources, we tested candidate markers identified in our antibody screen using the aforementioned hPSC retinal differentiation platform. We focused our efforts on biomarkers that had displayed robust staining patterns of well delineated retinal cell populations as we reasoned that too broadly or too narrowly expressed markers would not have the desired enrichment effect. In order to score the number of photoreceptors we chose a stringent CRX/RECOVERIN co‐expression signature; CRX is an early marker and by immunohistochemical analysis precedes RECOVERIN in human fetal photoreceptor cells (see Supporting Information Fig. S8). We did not rely solely on CRX labeling to identify photoreceptors as some reports indicate it is also expressed in retinal pigment epithelial cells 25, 26.

**Figure 3:**
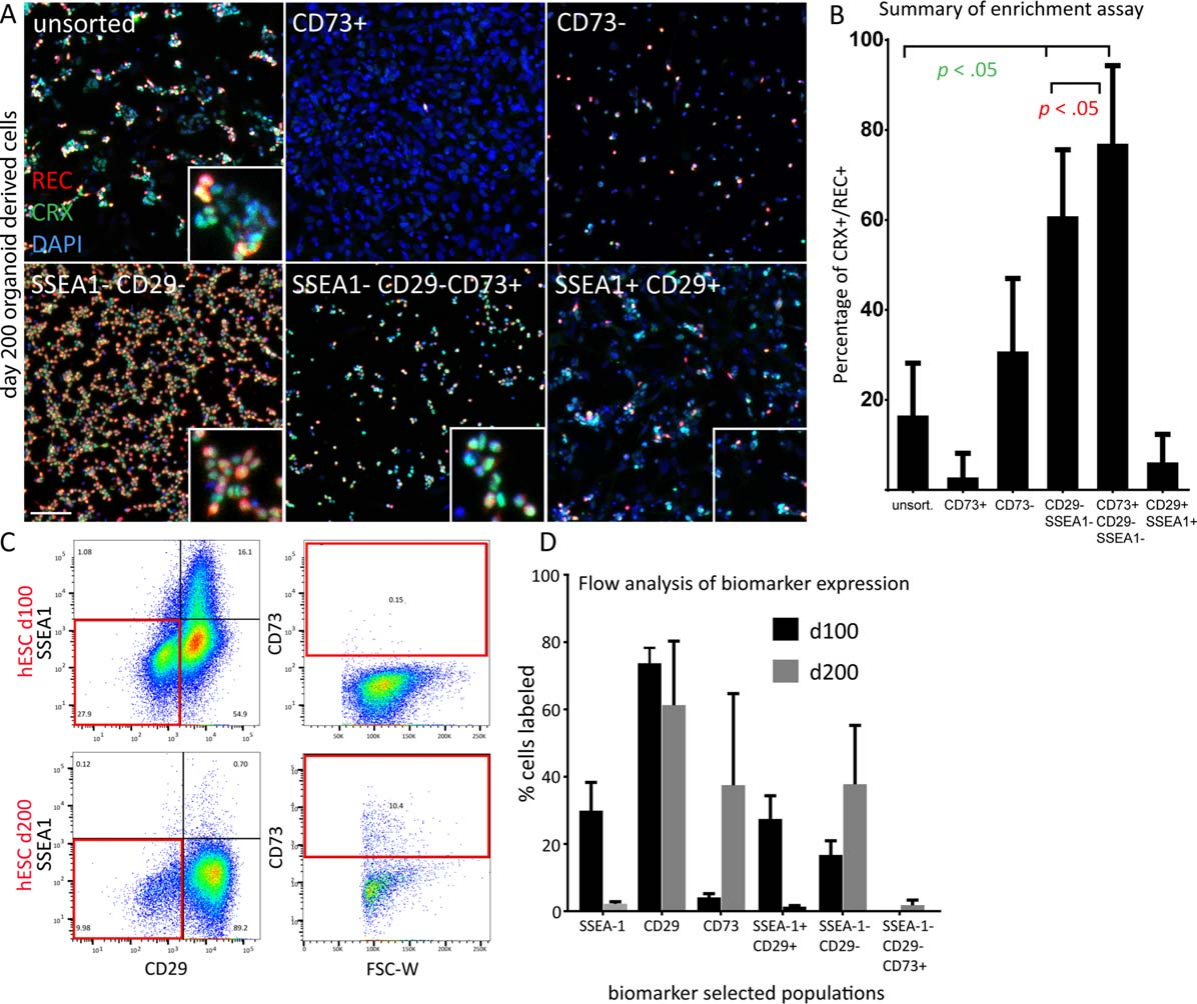
Photoreceptor enrichment assay using CD biomarkers identified in Lyoplate antibody screen. **(A, B)**: Immunocytochemical analysis of photoreceptor markers CRX and RECOVERIN on biomarker‐sorted retinal cells derived from human pluripotent stem cell differentiation cultures. Images in (A) show representative examples of cells derived from day 200 retinal organoids. Positive selection using CD73 alone fails to enrich photoreceptors (2.7% ± 5.3%, *n* = 8), while double negative selection by SSEA‐1 and CD29 alone, or followed by positive selection using CD73, efficiently enriches CRX/REC positive photoreceptor cells (60.8% ± 14.7%, *n* = 11 and 76.9% ± 17%, *n* = 3, respectively, compared with unsorted cells (16.5% ± 11.6%). (A, B). (B): Graph showing results of photoreceptor enrichment assay using biomarker combinations and displaying percentage of CRX/REC double positive photoreceptor cells after FAC‐sort. **(C)**: FAC‐sort gating strategy for using SSEA‐1, CD29, and CD73 in combination. Target gates are framed in red. **(D)**: Summary of flow cytometry analysis of biomarker expression at day 100 and day 200 of retinal organoid culture (*n* > 3). All values are shown as mean with corresponding standard deviation. ANOVA was used for statistical analysis. Scale bar 100 μm. Abbreviation: hESC, human embryonic stem cells.

Of the 16 candidate markers considered (GD2, CD29, SSEA‐1, SSEA‐4, CD9, CD73, CD133, EGFR, CD90, CD200, CD49f, CD147, CD184, CD107b, CD321, CD142), none showed any photoreceptor enrichment properties in our FAC‐sort based screening approach when used alone and for positive selection (data not shown). Interestingly, CD73, a biomarker previously described by our group 17, and others 27, 28, as a good tool for rod photoreceptor isolation in the mouse system was ineffective for this purpose using the hPSC‐derived retinal differentiation cultures (Fig. 3A, 3B). In fact, positive selection using CD73 resulted in a significant reduction of photoreceptors compared with the unsorted sample (2.7% ± 5.3% and 16.5% ± 11.6%, respectively, *n* > 3) suggesting CD73 labels nonphotoreceptor cells in our differentiation cultures. Similarly, FAC‐sorting for CD133, another biomarker with known expression in photoreceptors 16 did not yield higher numbers of photoreceptors post selection (CD133^+^: 19.6% ± 21%; CD133^‐^; 15% ± 14%; 16.5% ± 11% unsorted cells) (Supporting Information Fig. S3). However, we noticed that several biomarkers including CD29 and SSEA‐1 significantly enriched CRX/RECOVERIN expressing cells in their negative cell fractions after FAC‐sort (49.3% ± 18% and 35.6% ± 21% respectively, vs. 16.5% ± 11% unsorted cells, n > 9) (Supporting Information Fig. S3). CD29 always labeled the majority of cells in our differentiation system, whereas SSEA‐1 expression was more dynamic being expressed in 30% of cells at day 100 of differentiation and only in 2% of the cells at day 200 (Fig. 3D), explaining the superior photoreceptor enrichment properties displayed by negative cell selection using CD29 (Supporting Information Fig. S3). Using flow cytometry analysis, we observed that while SSEA‐1 expressing cells largely colabeled with CD29, a fraction of SSEA‐1 positive cells were consistently CD29 negative, indicating a distinct cell population. Therefore, we next tested if combining CD29 and SSEA‐1 for double negative cell selection using FAC‐sorting would lead to robust enrichment of CRX/RECOVERIN positive photoreceptors from retinal organoid cultures. Using this approach, we saw increased enrichment of photoreceptors in the CD29‐/SSEA‐1‐ (double negative) population (60.8% ± 14%) (Fig. 3A, 3B). On the other hand, CD29/SSEA‐1 double positive cell fractions were significantly depleted of photoreceptors compared with unsorted samples (6.1% ± 6% vs. 16.5% ± 11%) (Fig. 3A, 3B).

Last, we hypothesized that while CD73 on its own was not able to increase photoreceptor yields, in combination with CD29/SSEA‐1 based double negative selection it may increase photoreceptor enrichment from stem cell differentiation cultures as most of the nonphotoreceptor cells would be removed by the two markers. We found that 0.1%–1.5% of the cell population at day 100 and 200 showed a CD29‐/SSEA1‐/CD73+ profile, where CD73 positive cells made up 5% and 35% of the total cell population at day 100 and 200, respectively, (Fig. 3C, 3D). Addition of the positive selection step using CD73 (CD73+/CD29‐/SSEA1‐) to the purification protocol, yielded a higher photoreceptor enrichment (77% ± 17%) (Fig. 3A, 3B) compared with CD29 alone (49.3% ± 18%) (Supporting Information Fig. S3), but not significantly higher than CD29‐/SSEA‐1‐ double negative selection (60.8% ± 14%), which represents a cell fraction already highly enriched in photoreceptors. The cell selection mode utilizing all three biomarkers resulted in lower yields of CRX/RECOVERIN positive cells than CD29‐/SSEA‐1‐ double negative selection, as the number of CD73 expressing (CD73+/CD29‐/SSEA1‐) photoreceptors was relatively low in the culture system.

### Verification of Candidate Biomarkers in the Developing and Mature Human Retina

**Figure 4:**
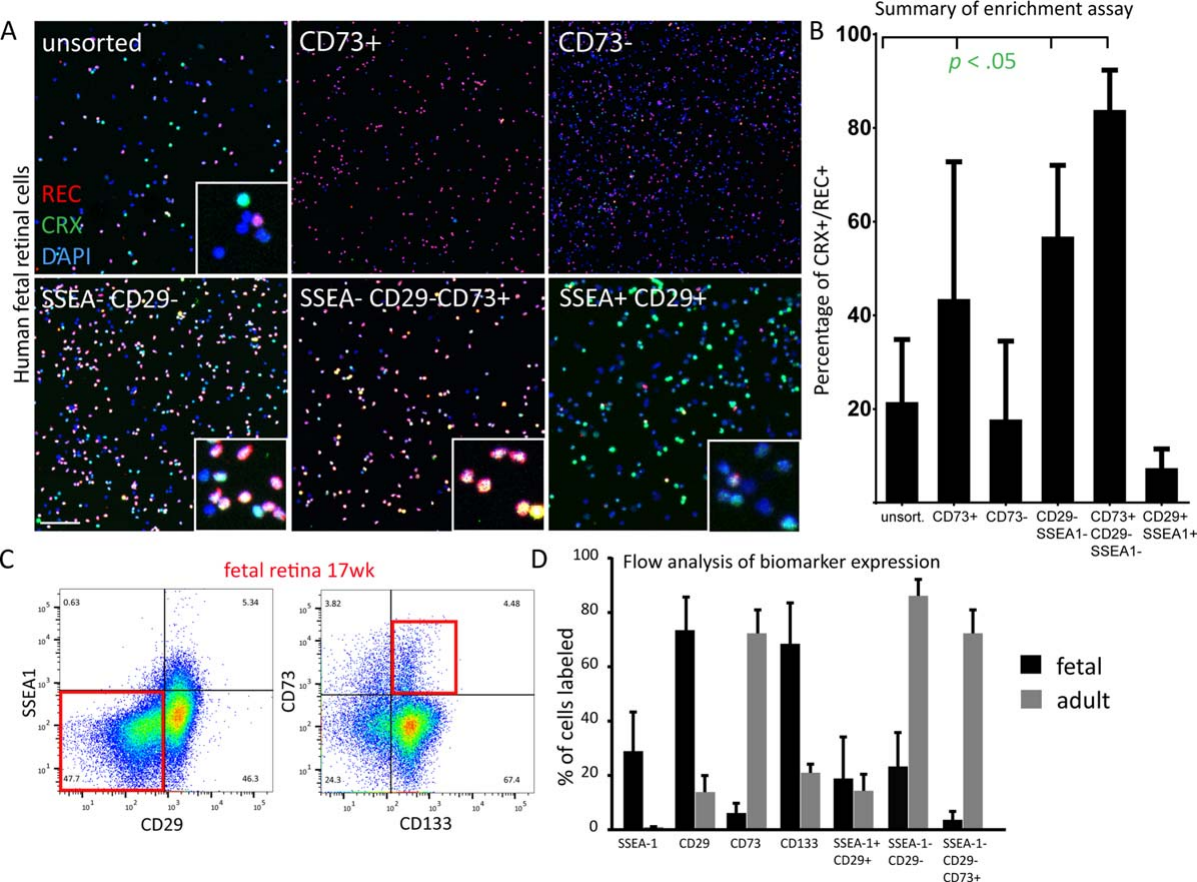
Photoreceptor enrichment assay using CD biomarkers identified in Lyoplate antibody screen. **(A, B)**: Immunocytochemical analysis of photoreceptor markers CRX and RECOVERIN on single biomarker sorted retinal cells derived from human fetal retinae (12–20 weeks gestation). Images in (A) show representative examples of cells derived from human fetal retina. Cell sorting based on the combination of SSEA‐1 and CD29 for double negative selection, or in conjunction with CD73 positive selection, leads to a significant enrichment of photoreceptor precursors from the developing human retina (56.8% ± 15.2%, *n* = 7 and 84.5% ± 8.2%, *n* = 8 respectively; Mean ± SD compared with unsorted (24% ± 14.2%). (B): Graph showing results of photoreceptor enrichment assay using biomarker combinations and displaying percentage of CRX/REC double positive photoreceptor cells after FAC‐sorting. CD73 sorting alone enriched photoreceptor cells to 42.1% ± 26% (*n* = 10) compared with unsorted cells (24% ± 14.2%; *n* = 13). **(C)**: Fluorescence‐activated cell sorting (FACS) gating strategy for using SSEA‐1, CD29 and CD73 in combination. Target gates are framed in red. **(D)**: Summary of flow cytometry analysis of biomarker expression using fetal (12–20 weeks) and adult human retinae (*n* > 3). All values are shown as mean with corresponding standard variation. ANOVA was used for statistical analysis. Scale bar 100 μm.

We also tested the candidate biomarkers for their ability to enrich immature photoreceptors from fetal human retinal tissue aged 10–22 PCW. In contrast to the hPSC‐derived organoid culture system, neither CD29 nor SSEA‐1 negative selection alone resulted in significant enrichment of CRX/RECOVERIN positive photoreceptors post FAC‐sorting (14.6% ± 2.2% and 13.6% ± 1.7%, respectively vs. 24% ± 14% unsorted; *n* = 13) (Supporting Information Fig. S4). By contrast, FAC‐sorting for CD73 enriched for photoreceptor cells, although with large variability between different fetal samples (Fig. 4B) (42.1% ± 26%; *n* = 10). We compared the CD73 photoreceptor enrichment capabilities at early and late stages of fetal development. While CD73+ positive cells selected from samples up to 12 weeks of gestation did not contain high proportions of photoreceptors (21.6% ± 4%), CD73+ cells isolated from samples older than 14 weeks of gestation showed significantly higher numbers of CRX/RECOVERIN positive photoreceptors (50.9% ± 26%) compared with unsorted retinal samples (24% ± 14%) (Supporting Information Fig. S5), consistent with onset of CD73 at later stages of photoreceptor differentiation. Flow cytometry analysis of biomarker expression showed that CD73 labeled less than 5% of the retinal cells during the 10–22 weeks developmental period compared with >70% in the adult retina and we similarly observed increased prevalence of cells with the CD29‐/SSEA1‐/CD73+ profile in the adult compared with the fetal retina (Fig. 4D).

Like in the hPSC differentiation system, CD29‐/SSEA‐1‐ double negative selection with, or without, additional CD73+ positive selection yielded higher photoreceptor yields from this cell source. Combination of all three biomarkers was superior in terms of photoreceptor cell enrichment compared with double negative selection using CD29/SSEA‐1 alone (84.5 ± 8.2 vs. 56.8 ± 15.2, respectively), (Fig. 4A, 4B). Photoreceptors enriched using the CD73+/CD29‐/CD15‐ signature expressed mostly RHODOPSIN (96.1 ± 1 of total cells collected vs. 40.4 ± 8.8 unsorted control) (Supporting Information Fig. S6) while no cone cell enrichment was observed (data not shown).

### Removal of Mitotically Active Cells via Biomarker Selection

**Figure 5:**
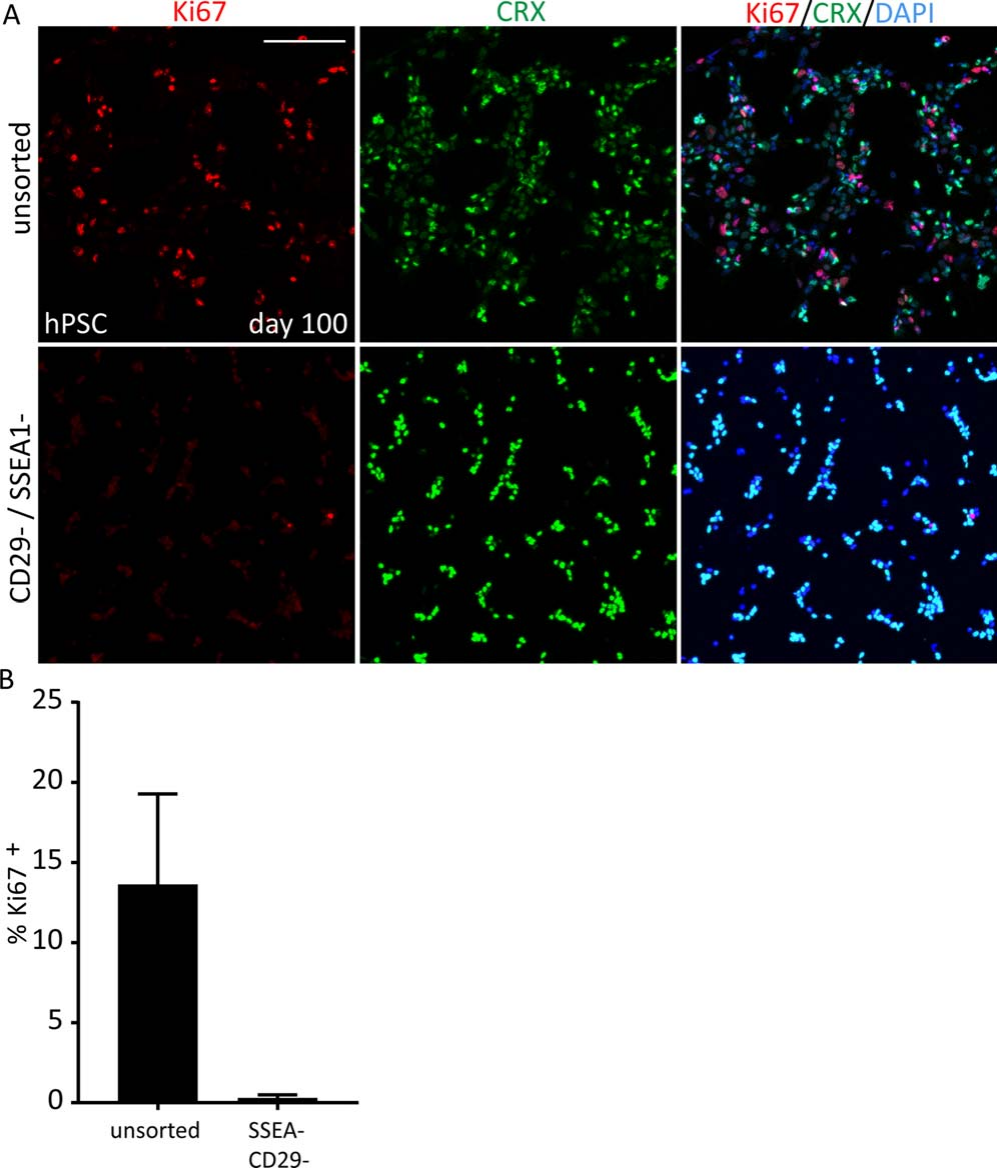
Removal of mitotically active cells from hPSC‐derived donor cell preparations using SSEA‐1/CD29 double negative cell selection. (A) Immunofluorescence analysis for cell cycle marker Ki67 and photoreceptor marker CRX on biomarker sorted cells. (B) Graph showing analysis of percentage of Ki67 cells in unsorted and SSEA1‐/CD29‐ cells. At day 100 of retinal differentiation in hPSC‐derived cultures 13.6% ± 5.6% of cells are mitotically active. After cell sorting using SSEA‐1 and CD29, almost all dividing cells have been eliminated from the cell mix (0.2% ± 0.19%). *n* = 3, H9 hESC line, Scale bar 100 μm. Abbreviation: hPSC, human pluripotent stem cell.

The inclusion of proliferative cell populations such as undifferentiated pluripotent stem cells or uncharacterized multipotent progenitor cells in cell preparations for cell therapy applications present a high safety risk owing to their ability to divide rapidly and form tumors in the subretinal space of patients following transplantation. It is, therefore, important that any cell selection strategy would need to ensure that these cells be removed prior to transplantation into patients with retinal dystrophy, not only to exclude the risk of tumor formation but also to better facilitate synaptic connectivity of graft photoreceptors to the host bipolar cells. We tested the ability of our human biomarker panel to remove mitotically active cells from hPSC organoid cultures at day 100 of differentiation. In these experiments, 13.6% ± 5% of cells in the differentiation cultures stained positive for Ki67, a marker of actively dividing cells that were mutually exclusive with staining for the photoreceptor marker CRX (Fig. 5). FAC‐sorting using CD29/SSEA‐1 double negative selection alone was sufficient to remove 98% (0.29% ± 0.19%) of the mitotic cells from the cell suspension, while the remaining cells almost entirely expressed the CRX transcription factor. These data show that mitotically active cells can be efficiently eliminated from hPSC‐derived donor cell preparations, prior to transplantation, by using the combination of photoreceptor cell surface biomarkers for negative cell selection.

### Transplantation of Biomarker Sorted Cells into Wild‐Type and RD1 Mouse Retinae

**Figure 6:**
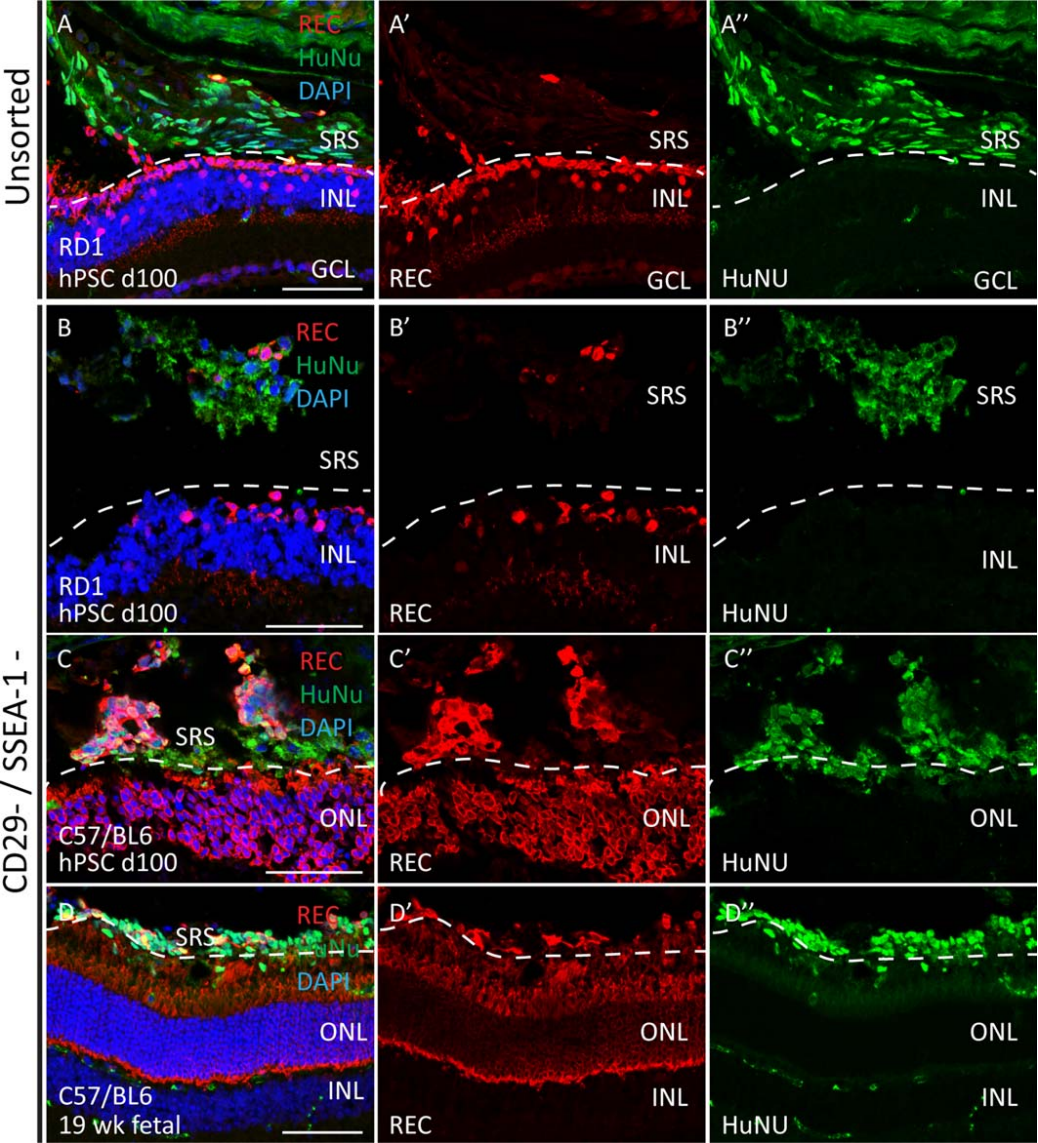
Survival of human biomarker sorted donor cells in the mouse retina. Unsorted or biomarker (CD29/SSEA‐1 –/–) sorted cells derived from day 100 hPSC organoid cultures or 19 weeks fetal retinae were injected subretinally into the eyes of wild‐type (C57/BL6) or RD1 mutant mice without immunosuppression **(A–D).** HuNu positive cells containing REC expressing photoreceptors were observed in the host retina after 7 days post transplantation. Donor cells were often observed aligning with host ONL in wild‐type, or with remaining INL cells in the RD1 retinae background. In wild‐type C57/BL6 recipient retina (C, D), a well formed outer nuclear layer containing HuNu negative, RECOVERIN positive photoreceptors is present. Degenerating retinae of 4‐week‐old RD1 mice (A, B), display at the most one row of RECOVERIN positive host photoreceptors (Fig. 6B). Abbreviations: GCL, ganglion cell layer; HuNu, human nuclei; INL, inner nuclear layer; ONL, outer nuclear layer; ONBL, outer neuroblastic layer; SRS, subretinal space. Scale bar 50 μm.

Previously, it was shown that heterogeneous cell preparations from both developing retina 29, but also hESC‐derived differentiation cultures 30, have the propensity to survive in the subretinal space of recipient mice post transplantation and exhibit variable photoreceptor marker expression. The ability of biomarker FAC‐sorted human photoreceptor populations to survive, not only the purification procedure, but also permanently establish themselves in the host retina is a critical hurdle toward developing a cell therapy approach for patients with retinal degenerations. To test viability, we performed a set of transplantations of unsorted dissociated cells and cells isolated via double negative CD29/SSEA‐1 selection from developing human fetal retinae and from day 100 hPSC differentiation cultures, into the sub‐retinal space of either C57/BL6 wild‐type, or an advanced model of degeneration, RD1 mutant mice. Triple selected populations were not used because cell yields from our standard protocols were lower than needed for transplantations experiments. Immunostaining of serial cryosections with an antibody directed against human nuclei or cytochrome oxidase C was used to detect human cells after transplantation (Supporting Information Fig. S7). Transplanted dissociated human fetal cells from 10, 11, 15, and 17 weeks retina survived in the SRS and a thick layer of human cells could be observed aligned with the wild‐type recipient ONL (Supporting Information Fig. S7A–S7I). Unsorted human fetal retinal cells directly injected (Supporting Information Fig. S7A–S7D), or cultured prior to use (Supporting Information Fig. S7E, S7F), gave rise to heterogeneous cell clusters in the SRS containing PAX6 positive interneurons and/or progenitors as well as RECOVERIN positive photoreceptor precursor cells (Supporting Information Fig. S7F, S7H, S7I). Transplants of CD29‐/SSEA1‐ selected cells from a 19‐weeks fetal retina into wild‐type retina (Fig. 6D) and CD29‐/SSEA1‐ selected cells from a 17‐week fetal retina into RD1 mice (Supporting Information Fig. S7J) either formed a layer of cells in the subretinal space aligned with the endogenous mouse rods and cones in the host outer nuclear layer or located as cell masses in the SRS (Fig. 6D and Supporting Information Fig. S7J). Recoverin‐positive human cells were readily detected in the fetal transplants (Fig. 6D and Supporting Information Fig. S7I). We also tested transplantation of dissociated day 100 ESC retinal cultures. After transplantation of unsorted ESC‐derived cells variably sized human cell clusters were detected around the transplantation site; Fig. 6A and Supporting Information Fig. S7K show views of human ESC‐derived cells directly juxtaposed to the host photoreceptor layer in both wild‐type and RD1 recipients with few RECOVERIN positive cells. Following transplantation of CD29‐/SSEA1‐ selected ESC‐derived cells in wild‐type C57/BL6 and RD1 recipient retina, human RECOVERIN positive photoreceptors cells were detected in the SRS either as cell clumps or aligned with the host ONL (Fig. 6B, 6C). Xenotransplant success was variable and precludes quantitative analysis. Supporting Information Figure S7L shows a trend toward increased RECOVERIN/HuNU double positive cells using sorted donor populations compared with unsorted populations from analysis of animals (*n* = 11) with successful xenografts. Taken together, these data indicate that biomarker selected, human photoreceptors can survive the stress associated with tissue dissociation, FAC‐sorting and sub‐retinal transplantation, and are able to persist in the sub‐retinal space of both normal and diseased retinae. Larger studies will be necessary to perform quantitative analysis of the performance of human xenograft cell transplants.

## Discussion

Cell replacement therapy is one of several promising future treatment options currently under intense investigation, the goal of which is the re‐introduction of healthy cone and rod photoreceptor cells into the degenerating patient retina. This approach may be particularly useful in cases where the endogenous photoreceptor population has already been severely decimated and other avenues such as gene therapy and neurotrophic protection are unlikely to be efficacious. We and others have shown previously that photoreceptor precursors can be introduced into the normal and degenerating mouse retina via subretinal injection and that transplanted cells are capable of improving visual function in mouse models of retinal degeneration 10, 15, 17, 18, 31, 32. Recent work has indicated that a new mechanism involving transfer of cytoplasmic material from transplanted cells to endogenous photoreceptors is important for rescue at early stages of degeneration 33, 34, 35. At the moment, the most feasible sources of donor cells for a future clinical application are human pluripotent stem cell cultures, in particular using three‐dimensional (3D) culture methods capable of generating complete in vitro generated retinae containing all relevant cell types. However, as donor cells are always produced alongside other cell populations, some with potentially detrimental properties, the successful translation of this approach to the clinic is dependent on the development of stringent cell selection and purification methods. For example, while inclusion in donor cell preparations of nonphotoreceptors may prevent or hinder establishment of connectivity to host retinal circuitry, presence of mitotically active cell types poses the risk of tumor formation in the host retina after transplantation. To date, isolation of target cells from animal model systems has been carried out using cell type specific reporter constructs stably integrated into the host genome, or delivered via viral vectors. However, for clinical application, manipulation of the donor cell's genetic material is not desirable due to the risk of inadvertent disturbance of the host gene expression and toxicity of the reporter gene. In order to circumvent genetic manipulation of cells, but also to meet the need for rigorous cell selection we previously developed a panel of photoreceptor biomarkers that can be effectively used to isolate transplantation‐competent rod precursors from 3D retinal differentiation cultures of mESC 16. In the present study, we have extended this paradigm to the human system and describe a set of cell surface biomarkers which are useful for the isolation of human donor cells for a range of applications including disease modeling, assessment of drug screens, and cell replacement therapy to treat retinal dystrophies.

Conducting an unbiased antibody screen using BD‐Lyoplates, we identified a total of 46 CD biomarkers robustly expressed in the human retina at various stages of development, and further analyzed a subset of 16 that showed restricted expression to defined cell populations. Several of the identified biomarkers differed from those found in similar experiments conducted using mouse retinal tissue demonstrating a degree of evolutionary divergence between mouse and human. Our photoreceptor enrichment assays did not identify any single CD biomarker capable of enriching photoreceptors from hPSC‐derived retinal cultures when used as a positive selector. Interestingly, sorting for CD73, a biomarker that we, and others, previously described as an effective donor cell selector in mouse mESC differentiation cultures 16, 27, 28 failed to purify photoreceptor cells in our experiments. This observation is likely due to the more wide spread expression of this marker in the human differentiation system and/or a relatively late onset of expression in photoreceptors compared with the rodent systems. While we have shown previously that CD73 is specific to mature photoreceptors in the adult human retina 16, it remains unclear when and if the majority of photoreceptors acquire its expression. During the developmental time window available for fetal tissue collection (8–22 weeks post conception) very few cells express this epitope, while our flow cytometry analysis shows that ∼70% of all retinal cells express CD73 in the adult retina. This correlates well with the overall number of rod photoreceptors in the adult and also mirrors our preferred photoreceptor biomarker signature (CD73+/CD29‐/SSEA1‐). Although from 14 weeks of gestation onward, we observed that CD73+ selected cells contained more photoreceptors than unsorted controls, overall we found that this marker is not a reliable tool for photoreceptor cell isolation when used alone. Our screen, however, did reveal a number of biomarkers that depleted donor cells, among them CD29 and SSEA‐1. While we have previously used SSEA‐1 for cell depletion in the mouse systems 16, CD29 is a novel negative selection tool in the context of the retina.

Our data shows that CD29 is the main driver behind photoreceptor enrichment when used for cell depletion, and combined with SSEA‐1 as a second negative selector or with CD73 for positive selection confers higher photoreceptor purity, albeit with lower overall yields. The current dual selection method labels an average of 16.75% of day 100 cultures and 44.5% of day 200 cultures (Fig. 3D), which in principle yields 6.7 to 17.8 million cells starting from a 6 well culture plate containing approximately 40 million cells. Yields most likely reflect variations of differentiation culture outputs at different time points and, in the context of fetal retinae, physiological differences between samples. In general, the combination of several biomarkers for cell isolation is considered to be superior compared with single markers due to the promiscuity of most known CD markers. In addition, negative selection is favorable for the development of good manufacturing practices (GMP) and avoids antibody carry over. In our experiments, use of the human biomarker combination allowed for photoreceptor cell purities of more than 80%, using colabeling of CRX and RECOVERIN to measure cell purity. Although the proportion of CRX singly positive cells was even higher in most cases, the fact that this transcription factor may also be expressed in other nonphotoreceptor cells, for example, RPE, bipolar cells, 25, 26 restricts its usefulness. Focusing our analysis on CRX/RECOVERIN double positive cells may therefore underestimate overall target cell enrichments. In our proof of principle experiments, we demonstrate that double negative cell selection alone based on CD29 and SSEA‐1 also reduces the number of Ki67 positive, dividing cells by more than 63‐fold. Positive selection using CD73 or addition of alternative negative selection markers identified in our screen might add further stringency for removal of these cells from future donor cell preparations.

Last, we demonstrate that biomarker sorted photoreceptor cells are viable and suitable for cell transplantation into both wild‐type and degenerating mouse retinae. We found that sorted graft cells survived the physiological stress associated with the purification and subretinal injection procedure forming photoreceptor rich clusters or layers of cells in the host retina adjacent to the endogenous retinal architecture. In these experiments, we used a human specific antibody to label the transplanted cells and no evidence of host cell staining using this antibody was observed. A significantly larger study will be required in the future to assess and optimize xenotransplantation outcomes and differential cell survival in different environments and to identify the numerous poorly understood factors that influence transplant success.

## Conclusion

The biomarker panel presented in this study enables for the first time, the enrichment of human photoreceptor cells from both developing retina and more importantly hPSC‐derived retinal organoid cultures. Features like cell purity, removal of mitotically active cells, and transplantability are comparable to previously described donor cell isolation methods developed in the mouse model system. Optimization of this approach for the use in a GMP‐grade environment will enable future therapeutic testing. Last, the usefulness of this facile photoreceptor isolation method extends to all circumstances in which high cell purities are required in the research setting such as transcriptomic and proteomic analyses for disease modeling or measuring drug‐induced changes in compound screens.

## Author Contributions

J.L. and E.W.: conception and design, collection and/or assembly of data, data analysis and interpretation, manuscript writing, financial support; D.B. and F.D.M.: collection and/or assembly of data, data analysis and interpretation; V.D.F. and J.W.B.B.: collection and/or assembly of data; K.W. and D.M.G.: training on the retinal differentiation protocol, manuscript writing; R.R.A.: conception and design, manuscript writing, financial support; J.C.S.: conception and design, data analysis and interpretation, manuscript writing, financial support, final approval of manuscript.

## Disclosure of Potential Conflicts of Interest

K.W. is employed at Cellular Dynamics International. D.G. discloses employment as Chief Scientific Officer, Opsis Therapeutics; a patent holder on 3D optic vesicles derived from hPSCs and ownership interest in Opsis Therapeutics. JCS, RRA JL and EW disclos Intellectual property rights/Inventor or patent holder for UCL Business PLC (patent application based on this work). The other authors indicated no potential conflicts of interest.

## Supporting information

Supplementary_Figure 1

Supplementary_Figure 2

Supplementary_Figure 3

Supplementary_Figure 4

Supplementary_Figure 5

Supplementary_Figure 6

Supplementary_Figure 7

Supplementary_Figure 8
