# Supplementary figures and images for: Isolation of Human Photoreceptor Precursors via a Cell Surface Marker Panel from Stem Cell‐Derived Retinal Organoids and Fetal Retinae
Source: Stem Cells. 2018 Feb 1;36(5):709–22. doi: 10.1002/stem.2775 (PMC5947711; doi:10.1002/stem.2775)

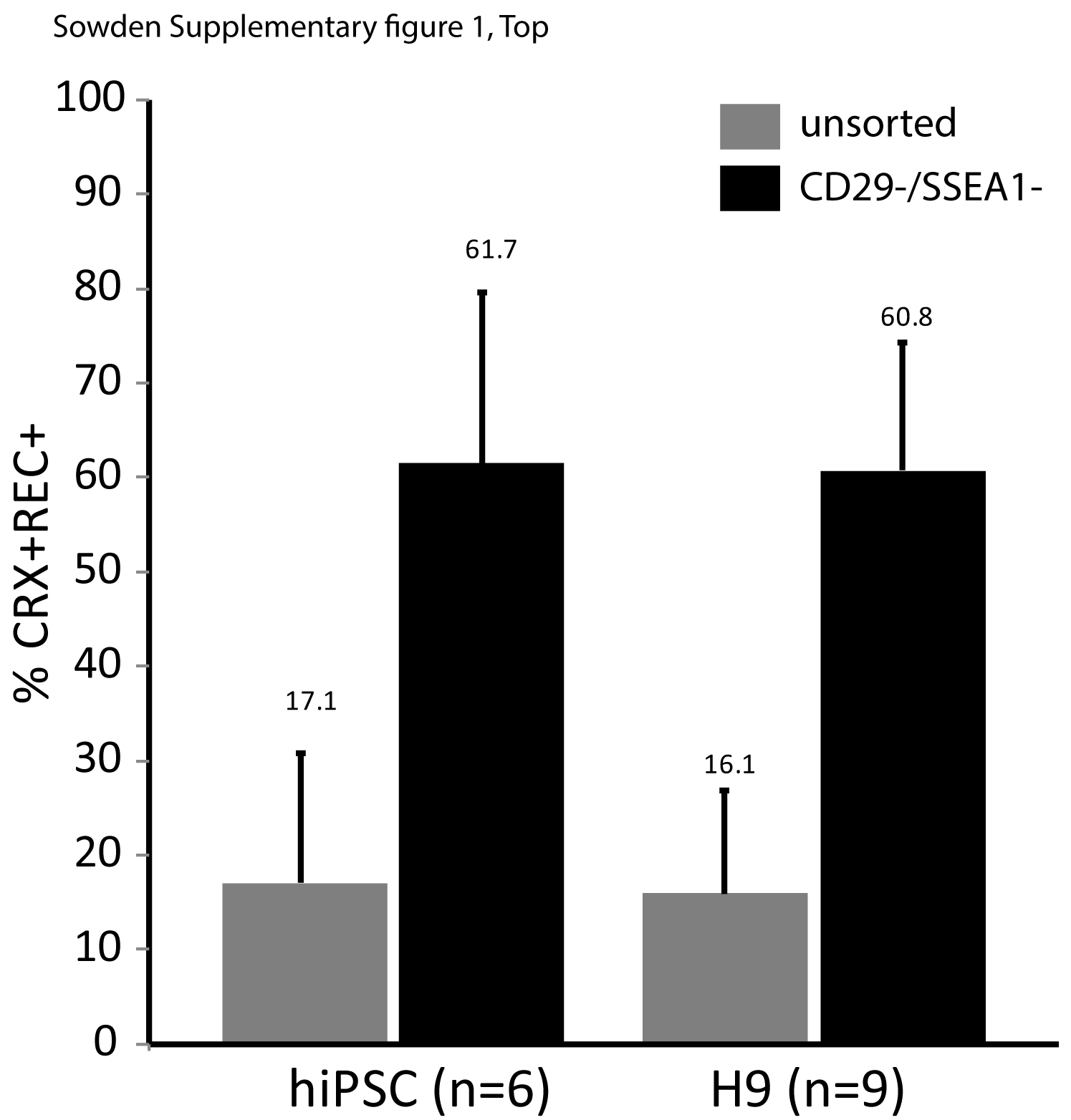

Supplement: Supplementary file 1 — Supplementary_Figure 1 [file STEM-36-709-s001.tif]

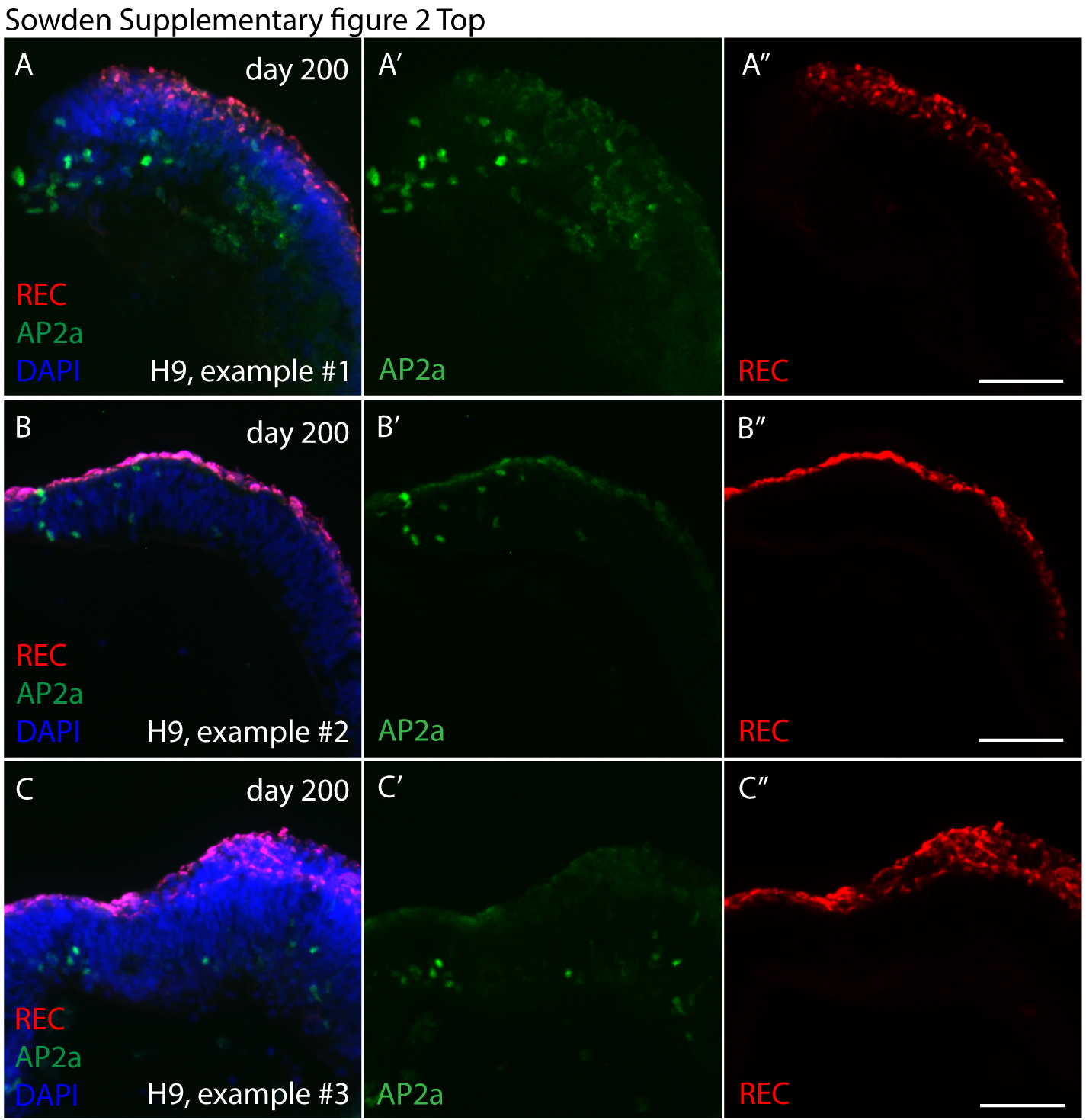

Supplement: Supplementary file 2 — Supplementary_Figure 2 [file STEM-36-709-s002.tif]

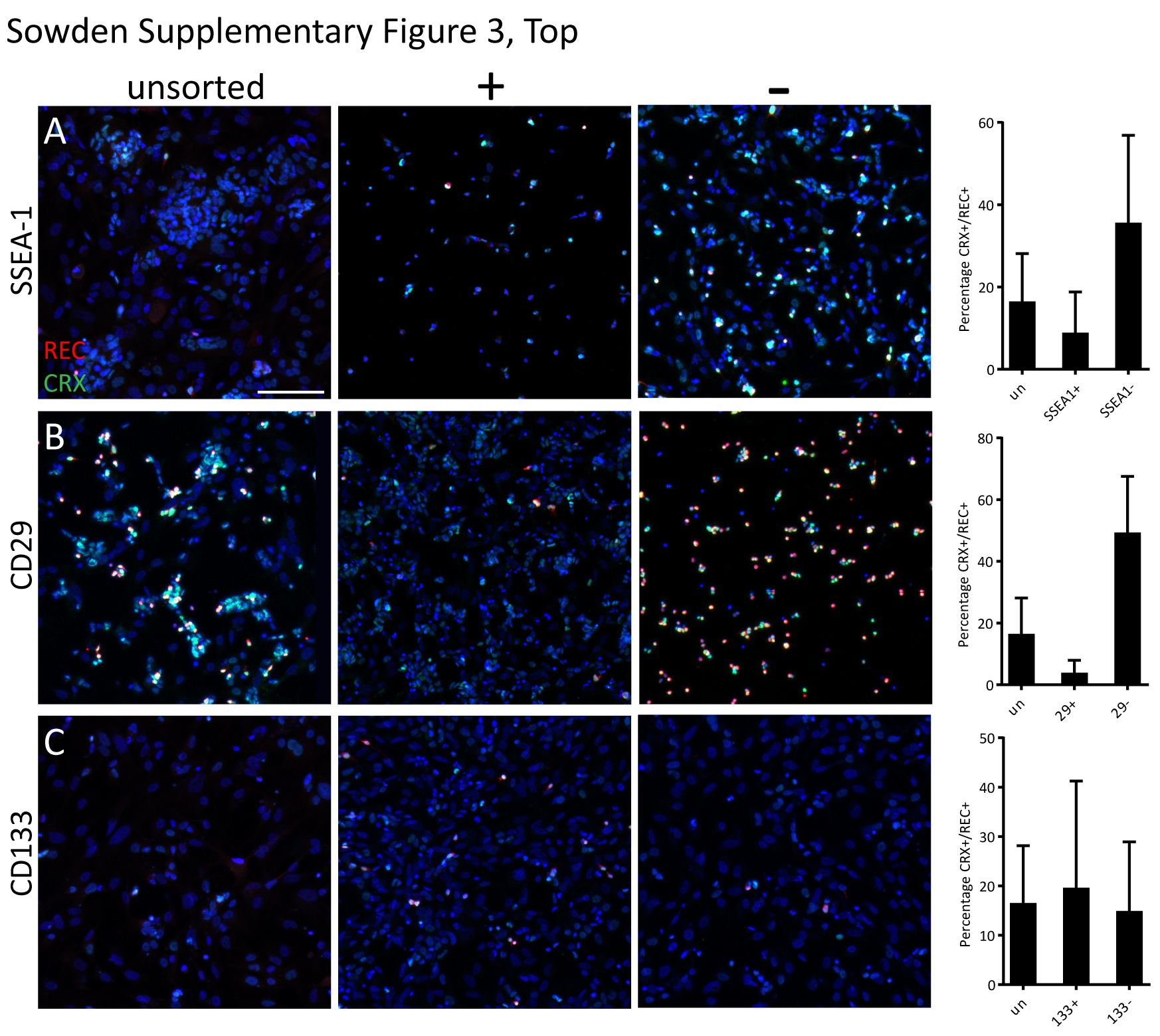

Supplement: Supplementary file 3 — Supplementary_Figure 3 [file STEM-36-709-s003.tif]

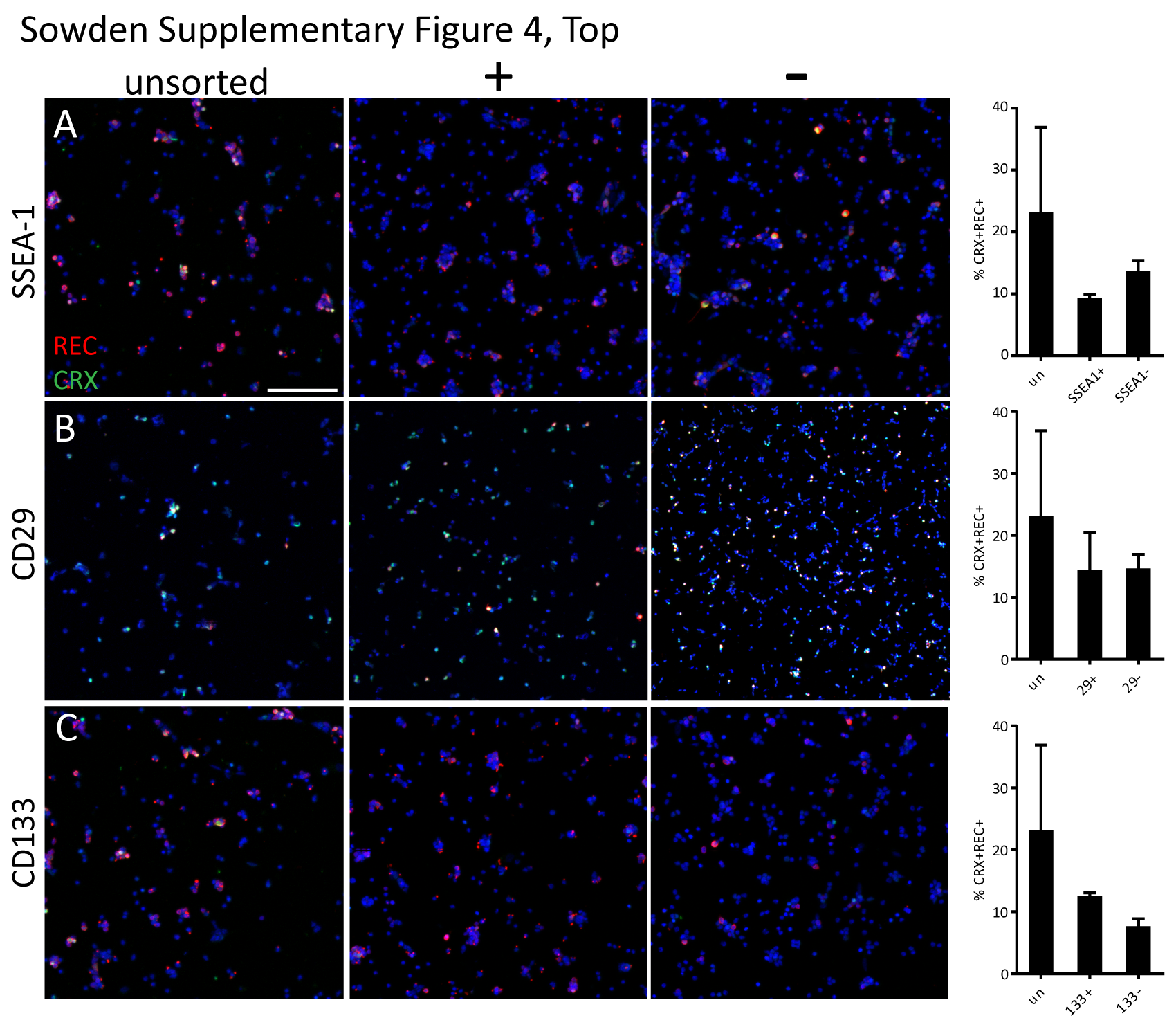

Supplement: Supplementary file 4 — Supplementary_Figure 4 [file STEM-36-709-s004.tif]

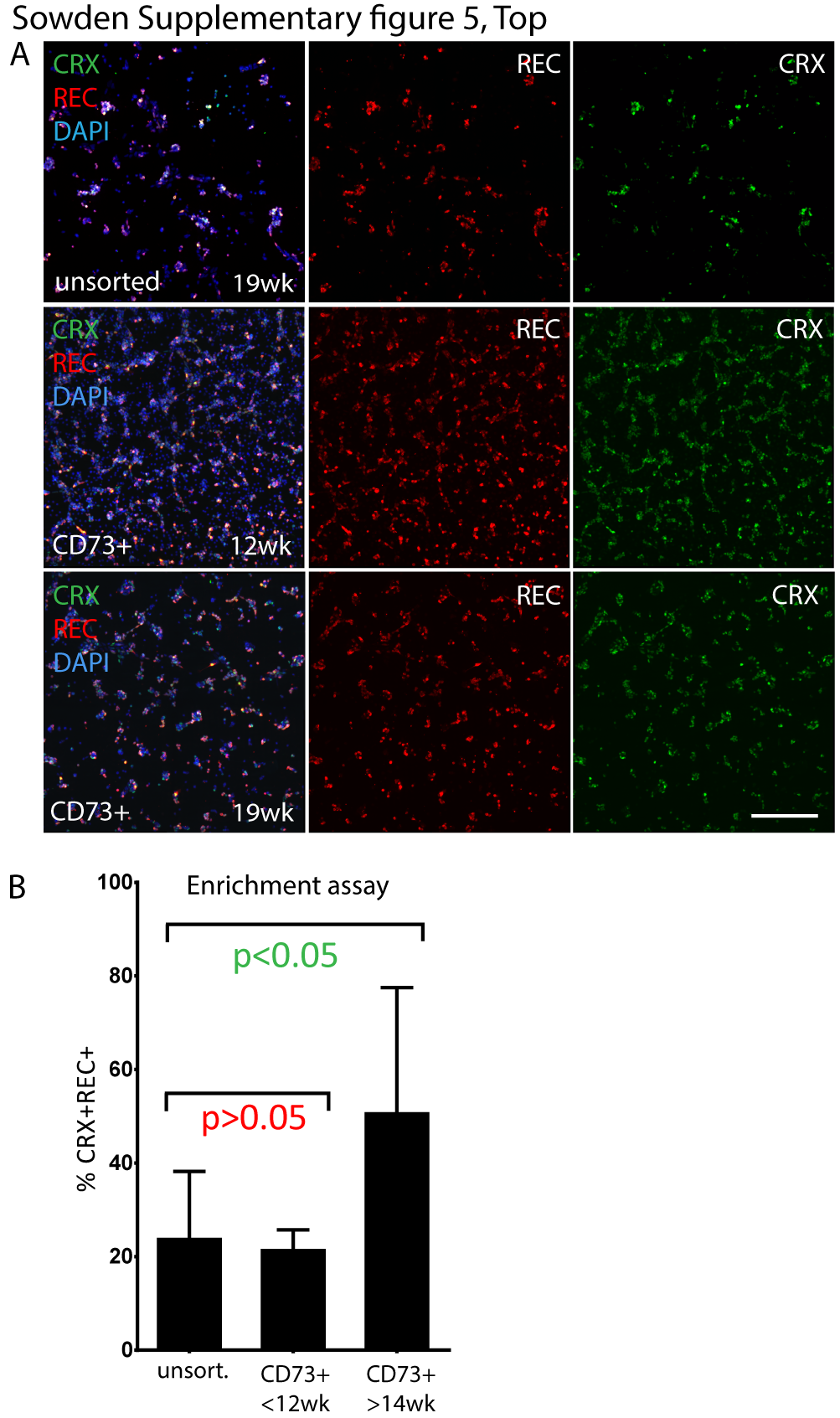

Supplement: Supplementary file 5 — Supplementary_Figure 5 [file STEM-36-709-s005.tif]

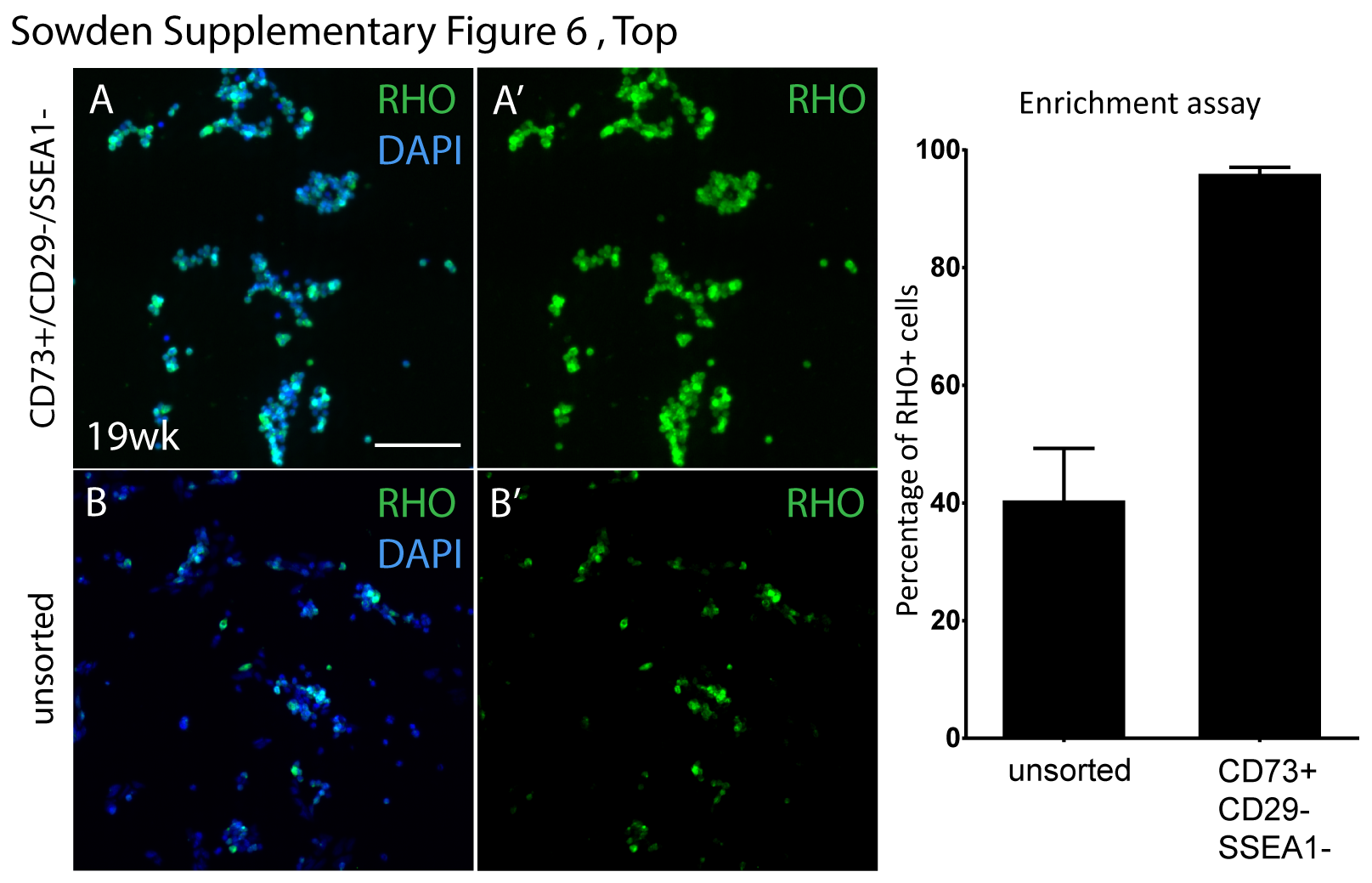

Supplement: Supplementary file 6 — Supplementary_Figure 6 [file STEM-36-709-s006.tif]

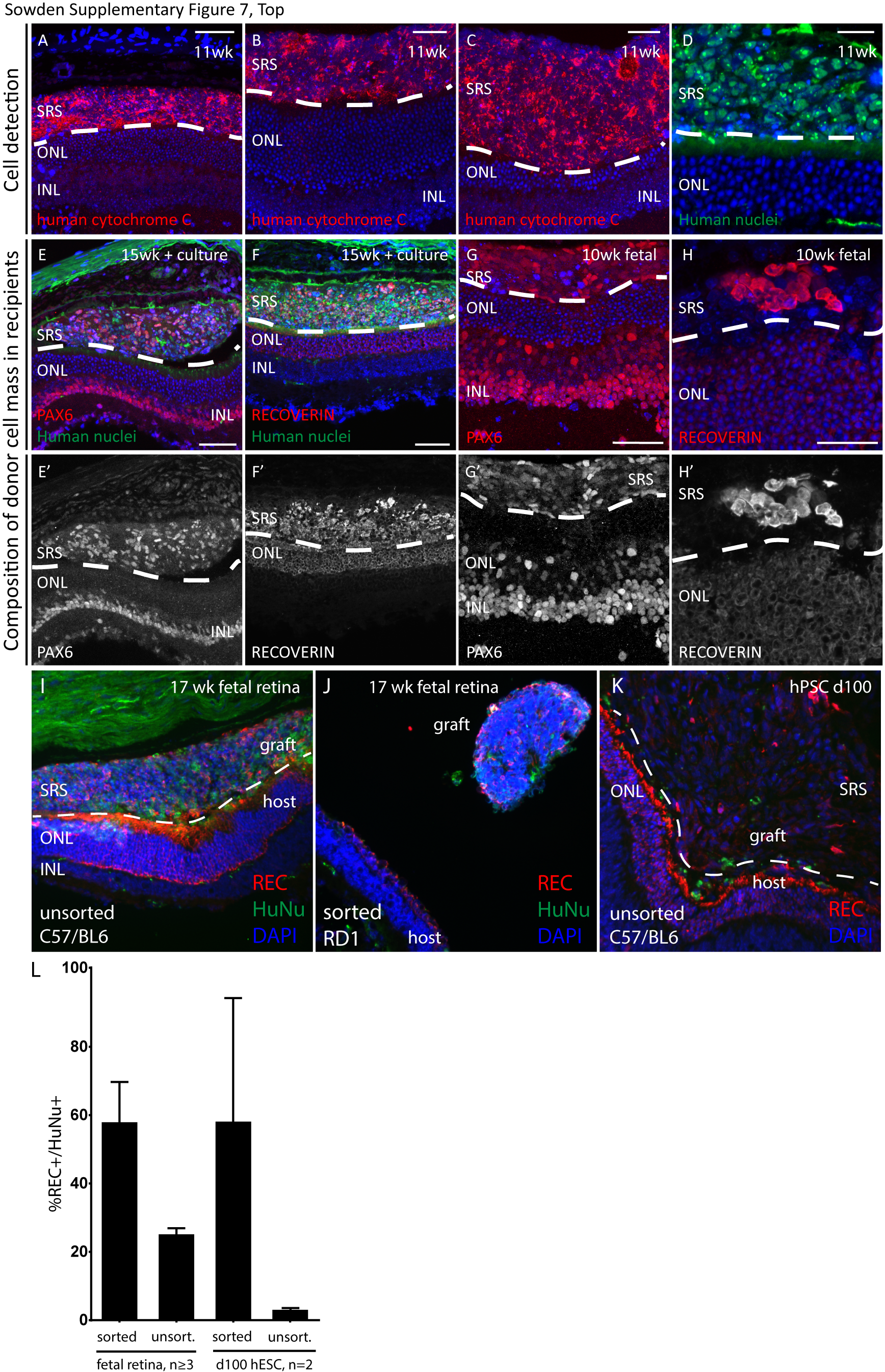

Supplement: Supplementary file 7 — Supplementary_Figure 7 [file STEM-36-709-s007.tiff]

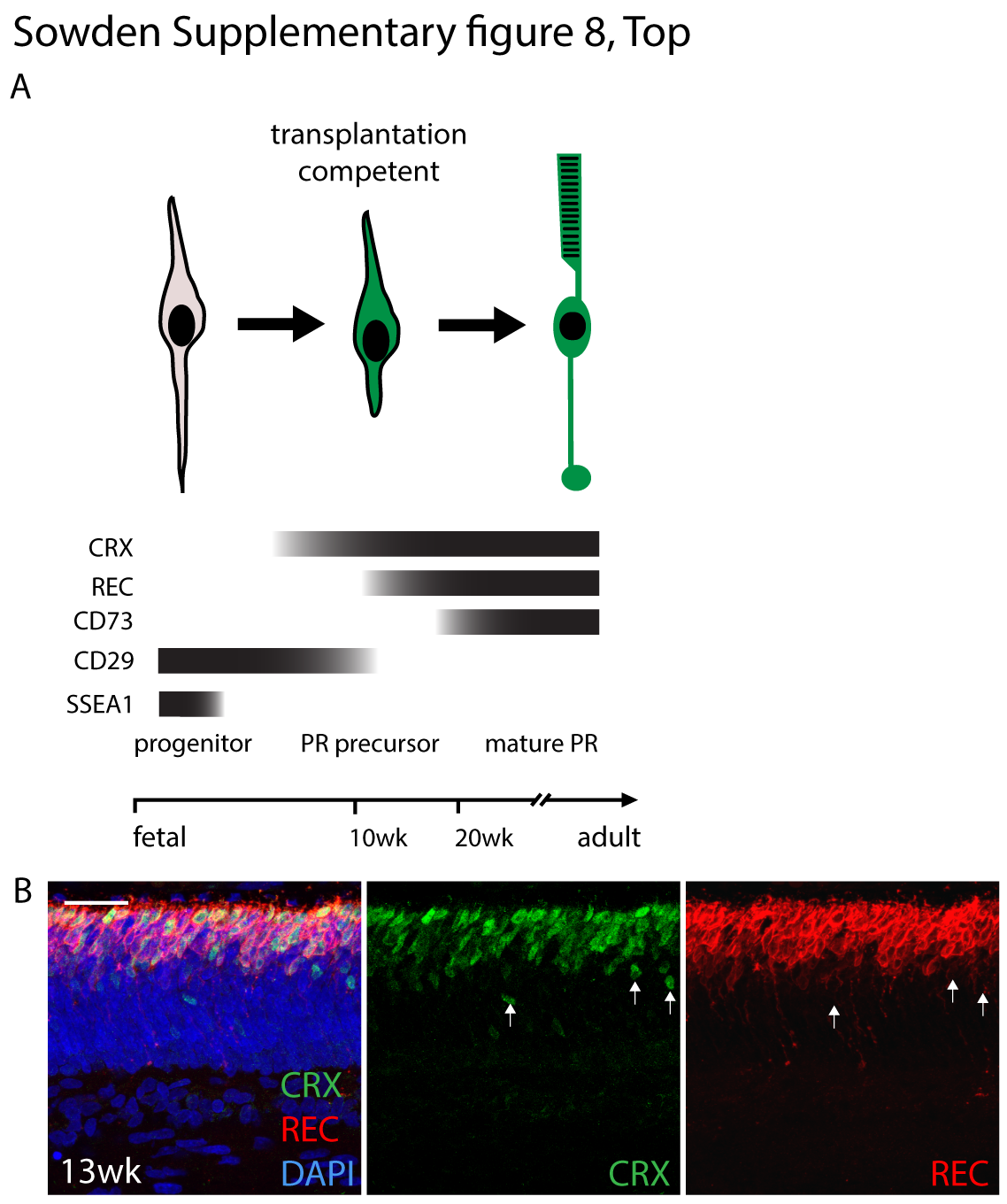

Supplement: Supplementary file 8 — Supplementary_Figure 8 [file STEM-36-709-s008.tif]
